# Treatment practices, characteristics and outcome of immunoglobulin A nephropathy – a Swiss single center experience

**DOI:** 10.3389/fneph.2026.1648950

**Published:** 2026-02-27

**Authors:** Danny Thieny Taing, Bruno Vogt, Laila-Yasmin Mani

**Affiliations:** 1 Department of Nephrology and Hypertension, Inselspital, Bern University Hospital, University of Bern, Bern, Switzerland; 2 Department of Radiation Oncology, Kantonsspital Aarau, Aarau, Switzerland

**Keywords:** cohort analysis, end-stage kidney disease, glomerular filtration rate, glomerulonephritis, hematuria, IgA nephropathy, immunosuppression, proteinuria

## Abstract

**Introduction:**

Immunoglobulin A nephropathy (IgAN) is the most common primary glomerulonephritis worldwide. Geographic differences in disease course and treatment response are well recognized. The purpose of this analysis was to study clinical and histological characteristics, treatment practices and outcome of IgAN cases from a Swiss tertiary center.

**Methods:**

This retrospective cohort analysis identified 158 cases of adult biopsy-proven IgAN by chart review diagnosed between 1980 and 2016. Following detailed phenotyping, standard descriptive methods and univariate analysis were applied.

**Results:**

The majority of patients was male and of European ancestry. At diagnosis, mean estimated glomerular filtration rate (eGFR) was 55.7 ml/min/1.73 m^2^, mean proteinuria was 2.4 g/d and 69.9% of the patients were hypertensive. Clinical presentation varied according to age. Initial biopsies showed moderate to severe tubular atrophy and interstitial fibrosis (IFTA) in 29.1% and crescents in 36.7% of cases. Therapy included renin-angiotensin-aldosterone-inhibitors in 86.7% as well as immunosuppressive therapy in 46.8% including steroids and other immunosuppressive drugs (28.7%), mainly azathioprin. Outcome included 34.1% complete and 22.2% partial remissions, relapses in 32.0% of patients, while 43.0% of patients progressed to ESKD during follow-up (median 100.0 months). Recurrence rate after transplantation was 18.8%. Immunosuppressive therapy was more frequently used in patients with higher proteinuria level, higher hematuria grade, lower eGFR, more intense IgA and complement C3 staining and crescents. Predictors of progression were higher age, lower eGFR, higher proteinuria and blood pressure as well as crescents and higher extent of IFTA on the initial biopsy.

**Conclusions:**

This retrospective cohort analysis gives insight into characteristics and outcome of patients with IgAN from a Swiss tertiary center, treatment practices as well as predictors of outcome and therapy choices. A comparatively high use of immunosuppressive treatment including non-steroid-based regimens was found along with a high rate of progression to ESKD.

## 1 Introduction

Immunoglobulin A (IgA) nephropathy is the most common primary glomerulonephritis worldwide (1, 2). It is defined histologically by the dominant or co-dominant glomerular deposition of IgA1 antibodies in a mesangial location (3). Progression to end-stage kidney disease (ESKD) occurs in a substantial part of affected patients (4). However, the range of clinical manifestations is wide reaching from asymptomatic isolated urinary abnormalities, progressive chronic impairment of kidney function or nephrotic syndrome to rapid progressive acute kidney injury (AKI) and systemic vasculitis (Henoch-Schönlein purpura) (5, 6). Moreover, large geographic and ethnic differences exist with regard to disease prevalence, presentation, outcome, and response to therapy (5, 7–12). Taken together, these data suggest IgA nephropathy as a disease spectrum rather than a well-defined disease entity (13, 14).

The primary form of IgA nephropathy is an immune-mediated disease involving a multi-hit pathogenesis (15). Until recently, treatment approaches have focused on supportive care including renin-angiotensin-aldosterone-system (RAAS) inhibition, more recently sodium-glucose cotransporter-2 inhibition and optimal control of cardiovascular risk factors (16, 17). The role of an autoimmunity-oriented approach as opposed to a purely chronic kidney disease (CKD)-oriented approach has been controversially debated during the last decades (18, 19). Given the paucity of high-quality clinical data and concern for medication-induced toxicity, immunosuppressive treatment has generally been reserved for patients with high risk of disease progression or rapid progressive disease course (20). However, local therapy regimens varied greatly according to geographic location and probably even according to centers (21).

In this work, we aimed to perform a detailed review of all cases of patients with IgA nephropathy treated at a Swiss tertiary center with focus on disease characteristics, treatment practices and outcome as well as to evaluate potential predictors of therapeutic choices and patient outcome.

## 2 Materials and methods

The study was designed as retrospective cohort analysis including all adult patients with a diagnosis of biopsy-proven IgA nephropathy included in the electronic medical record (introduced in November 2008) of the University clinic for Nephrology and Hypertension at the University Hospital Bern as of July 19th 2016. The electronic medical record database containing all patients followed or having been followed at our center was searched using the term “IgA”. Inclusion criteria were patients with a diagnosis of IgA nephropathy older than 18 years old. Exclusion criteria were lack of performed kidney biopsy and lack of clinical data entries.

For eligible patients, chart review was performed by collecting relevant pre-defined disease-related data within the electronic medical records. The dataset contained the following parameters: Demographic parameters (age, sex as noted in the clinical chart, ethnicity, place of birth); family history (e.g. kidney disease, consanguinity); clinical parameters (height, body weight, body mass index, blood pressure); clinical presentation (renal manifestations: nephrotic syndrome, acute kidney injury and AKIN stage, CKD stage according to KDIGO, edema, flank pain and extrarenal manifestations: purpura, arthritis, abdominal pain); laboratory parameters (hemoglobin, serum albumin, serum creatinine, estimated glomerular filtration rate (eGFR) using Chronic Kidney Disease Epidemiology Collaboration (CKD-EPI) 2009 formula, CKD stage according to Kidney Disease: Improving Global Outcomes, measured creatinine clearance, 24-hour urine protein, 24-hour urine albumin, urinary protein-creatinine ratio (g/mol), urinary albumin-creatinine ratio, hematuria intensity; proteinuria level was assessed using proteinuria as measured from an adequately collected 24h-urine collection if available or estimated from urinary protein-creatinine ratio); histological parameters (proportion of glomeruli with crescents, Haas classification (22) stage and Oxford classification/MEST score (23), tubular atrophy and interstitial fibrosis (IFTA) grading (according to original pathology report; degree of tubular atrophy/interstitial fibrosis (mild < 25%, moderate 25-50%, severe >50%)), IgA/IgG and complement C3 deposit grading; administered therapies (RAAS inhibitors, steroids, immunosuppressive drugs, tonsillectomy); outcome data (remission, ESKD, dialysis or kidney transplantation, recurrence of IgA nephropathy in the kidney transplant, death), as well as clinical and laboratory parameters at the time of last follow-up.

Complete remission was defined as sustained proteinuria < 0.3 g/d with preserved kidney function within one year after treatment start. Partial remission was defined as ≥ 50% decrease in proteinuria and proteinuria ≥0.3 g/d and <1 g/d within one year after treatment start. No remission was defined as not fulfilling criteria for remission. Relapse was defined as the reappearance of proteinuria >1g/d and increase of 50% from the lowest level of proteinuria in remission. Acute kidney injury was defined according to Kidney Disease Improving Global Outcomes Guidelines 2012 (24). Reduced GFR (rGFR) was defined as decrease in eGFR <60 ml/min/1.73 m^2^. There were no prevailing standard therapy guidelines and treatment was performed according to treating nephrologists.

Statistical analysis was performed using Microsoft Excel^®^ 2016 for standard descriptive methods (means, standard deviation or medians and ranges as appropriate). SPSS Version: 28.0.1.1 (14) was used to carry out group comparisons: Continuous variables were compared using student’s t-test on a two-sided significance level of 0.05. Dichotomic variables were compared using Chi square test on a two-sided significance level of 0.05.

The study was approved by the cantonal ethics committee of the canton of Bern, Switzerland (approval number 2022-02292).

## 3 Results

### 3.1 Patient cohort

**Figure 1:**
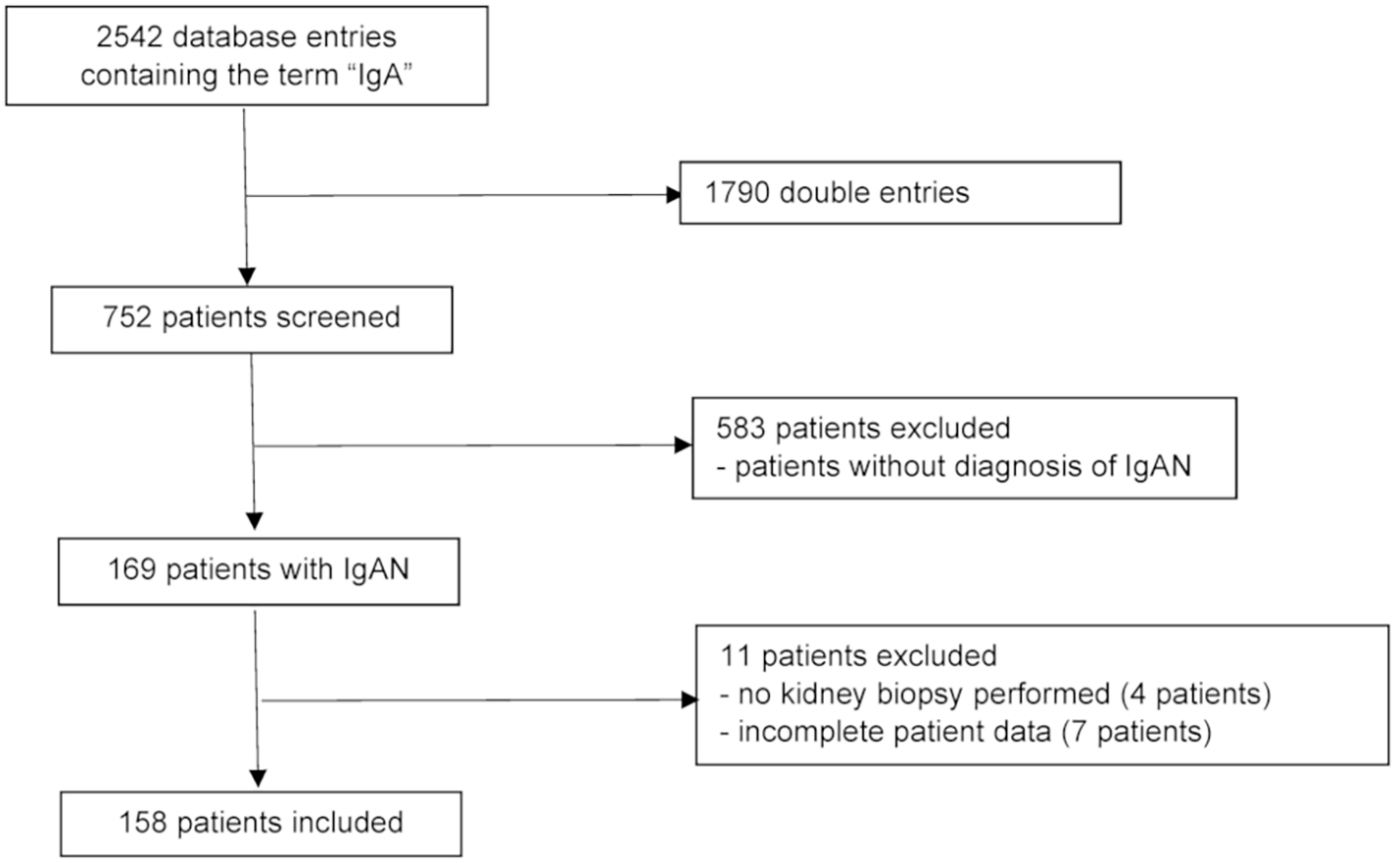
Patient flow chart. IgAN: IgA nephropathy.

The screening of the hospital’s electronic database for the term “IgA” between 1980 and 2016 resulted in 2542 patient entries. After exclusion of double entries, 752 patients were screened. Among those, 583 had no diagnosis of IgA nephropathy and were therefore excluded. Another 11 patients were excluded because of lack of histological confirmation or of missing clinical data. Finally, a total of 158 patients with IgA nephropathy diagnosed between 1980 and 2016 was included in the analysis (**Figure 1**).

### 3.2 Baseline characteristics, clinical and histological presentation

Baseline characteristics of the included patients are shown in **Table 1**. The majority of patients was male and of European ancestry. Positive family history for IgA nephropathy was present in 0.6% of patients.

**Table 1:** Baseline patient characteristics at diagnosis (n=158).

Demographics and baseline clinical data	
Age (years)	53.5 ± 14.8
Gender (%)	
- female	21.1
- male	78.9
Ethnicity (%)	
- European ancestry	85.4
- Asian	8.9
- African	3.2
- Hispanic	2.5
BMI (kg/m^2^)	26.7 ± 6.1
Positive family history for IgA nephropathy (%)	0.6

Clinical presentation	
Blood pressure (mmHg)	
- systolic (n=127)	138.3 ± 21.9
- diastolic (n=127)	84.0 ± 14.2
Hypertension (%) (n=109)	69.9
Serum creatinine (µmol/l) (n=136)	175.4 ± 126.7
Estimated glomerular filtration rate* (ml/min/1.73 m^2^) (n=136)	55.7 ± 30.6
Proteinuria^+^ (g/d) (n=132)	2.4 ± 2.0
Nephrotic syndrome (%)	9.5
Presence of microhematuria (%) (n=125)	90.4
Presence of macrohematuria (%) (n=153)	29.1
Extrarenal symptoms (%) (n=153)	4.58

Histological characteristics	
Presence of crescents (%) (n=151)	36.7
Presence of moderate to severe tubular atrophy and interstitial fibrosis^#^ (% of patients) (n=141)	29.1
MEST-C score^++^ (n=31) n (%)	
- mesangial hypercellularity	
M0	12 (38.7)
M1	19 (61.3)
- endocapillary hypercellularity	
E0	25 (80.6)
E1	6 (19.4)
- segmental glomerulosclerosis	
S0	13 (41.9)
S1	18 (58.1)
- tubular atrophy/interstitial fibrosis	
T0	22 (71.0)
T1	8 (25.8)
T2	1 (3.2)
- cellular/fibrocellular crescents	
C0	NA
C1	NA
C2	NA

Values are expressed as means with standard deviation or proportions. Extrarenal symptoms included purpura, arthritis and abdominal pain. *according to Chronic Kidney Disease Epidemiology Collaboration 2009 formula. ^+^measured from adequately collected 24h-urine collection if available or estimated from urinary protein-creatinine ratio.^#^ treated as binary variable. MEST-Score according to Oxford classification. ^++^MEST score available for a small subset of patients (n=31) due to diagnosis before introduction of the Oxford classification in the majority of patients.

At presentation, nearly one third of patients was symptomatic including macrohematuria (29.1%), edema (19.0%) or flank pain (9.5%). Roughly 5% of patients presented with extrarenal symptoms including purpura, arthritis or abdominal pain. The majority (69.9%) of patients was hypertensive. As expected, hematuria was seen in almost all patients, while over half of the patients presented with proteinuria ≥ 1 g/d with an overall mean proteinuria of 2.4 g/d. Almost one of ten patients had nephrotic syndrome at the initial presentation. Baseline kidney function ranged from preserved to severely impaired with a mean eGFR according to CKD-EPI across the cohort of 55.7 ml/min/1.73 m^2^. Three patients required dialysis treatment at presentation. Overall, most of the patients (86.7%) presented with chronically impaired kidney function.

**Figure 2:**
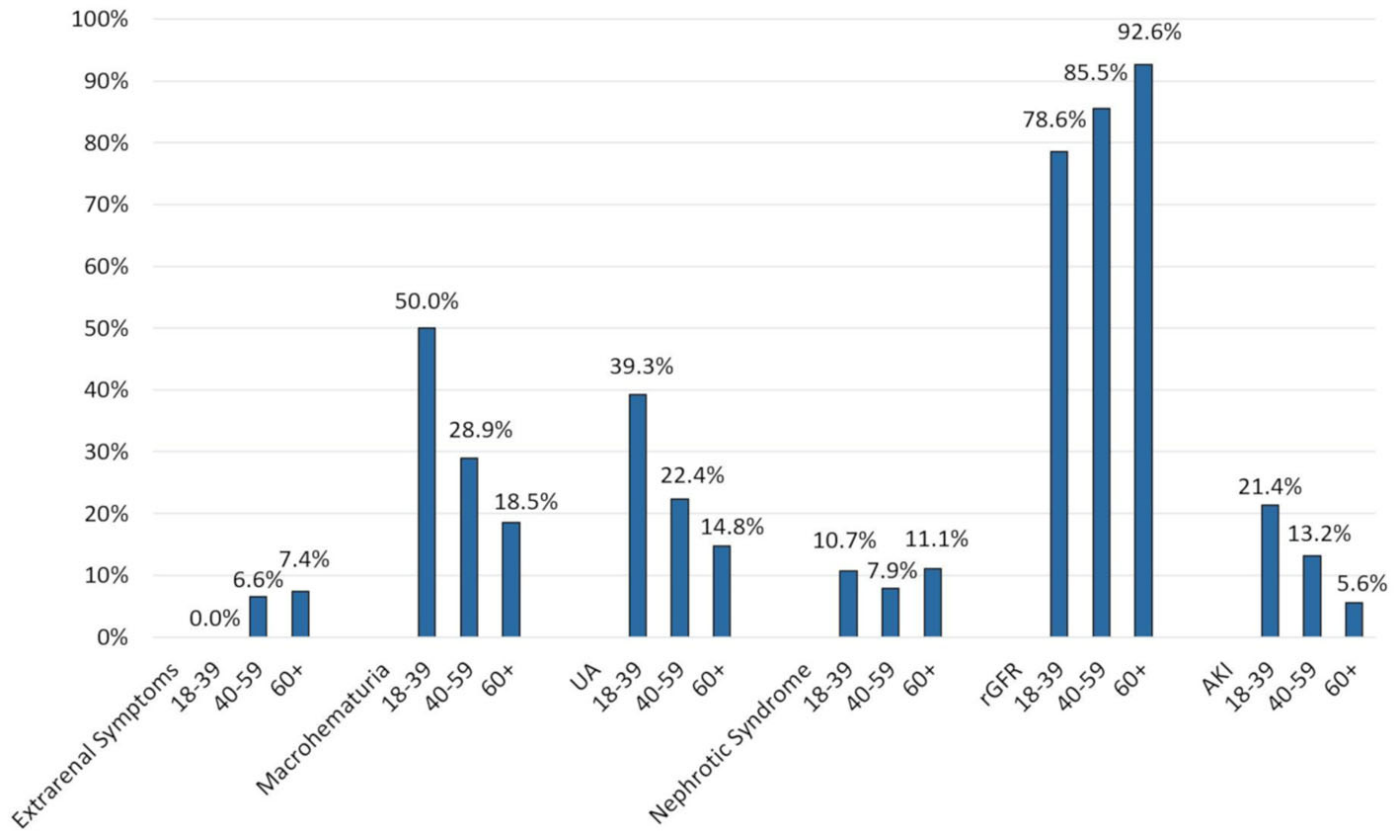
Clinical presentation according to age. Values are expressed as percentage of patients in each age group presenting with a particular clinical picture (mutually exclusive). Age groups: 18–39 years (n = 28), 40–59 years (n = 76), 60+ years (n = 54). Extrarenal symptoms included purpura, arthritis and abdominal pain. UA, Asymptomatic urine abnormalities; rGFR, reduced glomerular filtration rate (<60 ml/min/1.73 m^2^).

Clinical presentation varied according to age. Thus, younger patients (18–39 years) presented more frequently with macrohematuria and asymptomatic urine abnormalities and acute kidney injury at time of diagnosis while nephrotic syndrome and extrarenal symptoms were recorded in similar proportions of patients (**Figure 2**).

**Figure 3:**
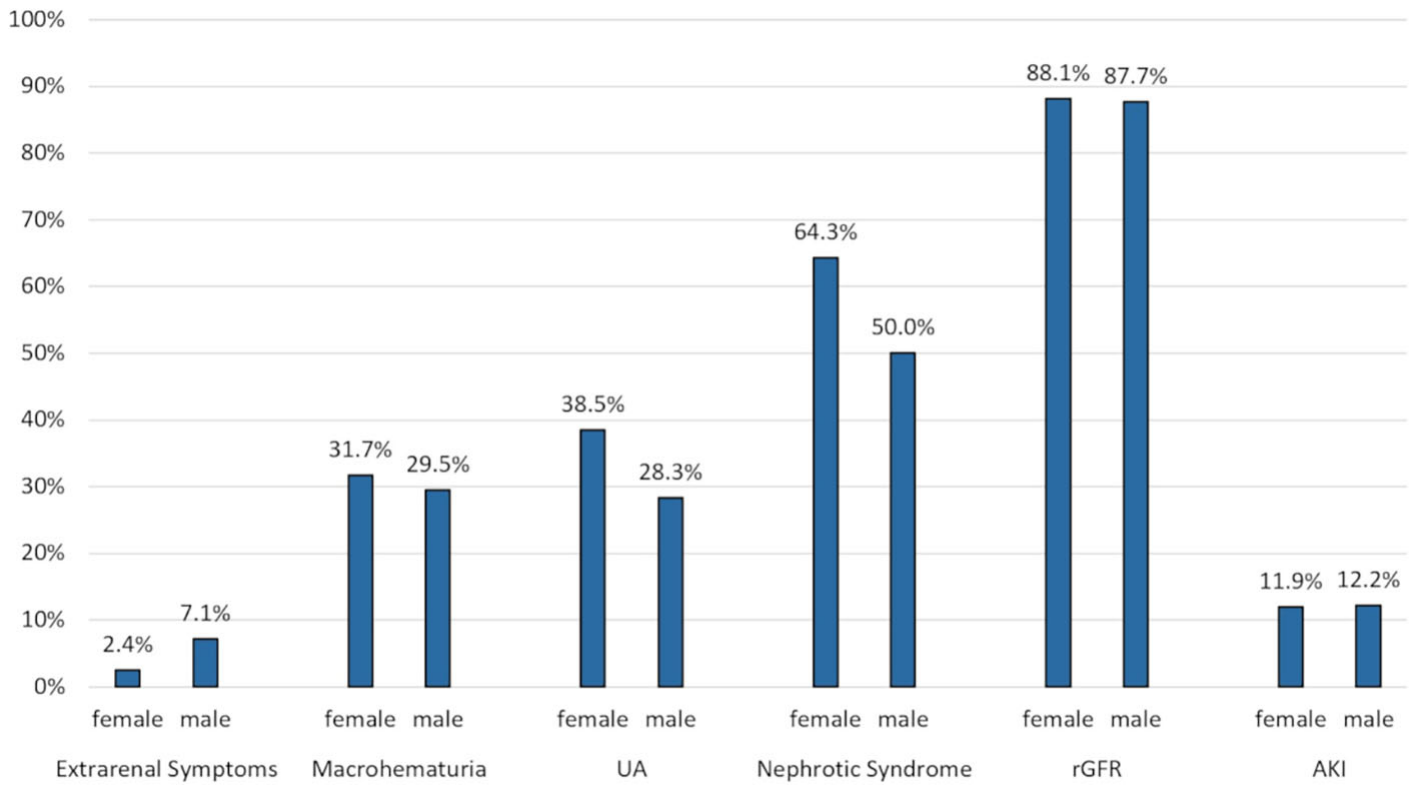
Clinical presentation according to sex. Values are expressed as percentage of patients in each age group presenting with a particular clinical picture (mutually exclusive). Extrarenal symptoms included purpura, arthritis and abdominal pain. UA, Asymptomatic urine abnormalities; rGFR, reduced glomerular filtration rate (<60 ml/min/1.73 m^2^).

Furthermore, clinical presentation varied according to sex. By trend, women presented more frequently with nephrotic syndrome and asymptomatic urine abnormalities whereas extrarenal symptoms tended to be more frequent in men (**Figure 3**).

The majority of kidney biopsies was analyzed before introduction of the Oxford classification. In 36.7% of biopsies, crescents were present; in 1.9% of biopsies, more than 50% of the glomeruli were affected by crescents. Moderate to severe tubular atrophy and interstitial fibrosis was noted in roughly a third of patients on the initial biopsy. MEST scores, where available, showed endocapillary hypercellularity in a minority of patients, yet low scores for tubular atrophy/interstitial fibrosis. There were no cases of concomitant diabetic nephropathy.

### 3.3 Treatment and outcome

**Figure 4:**
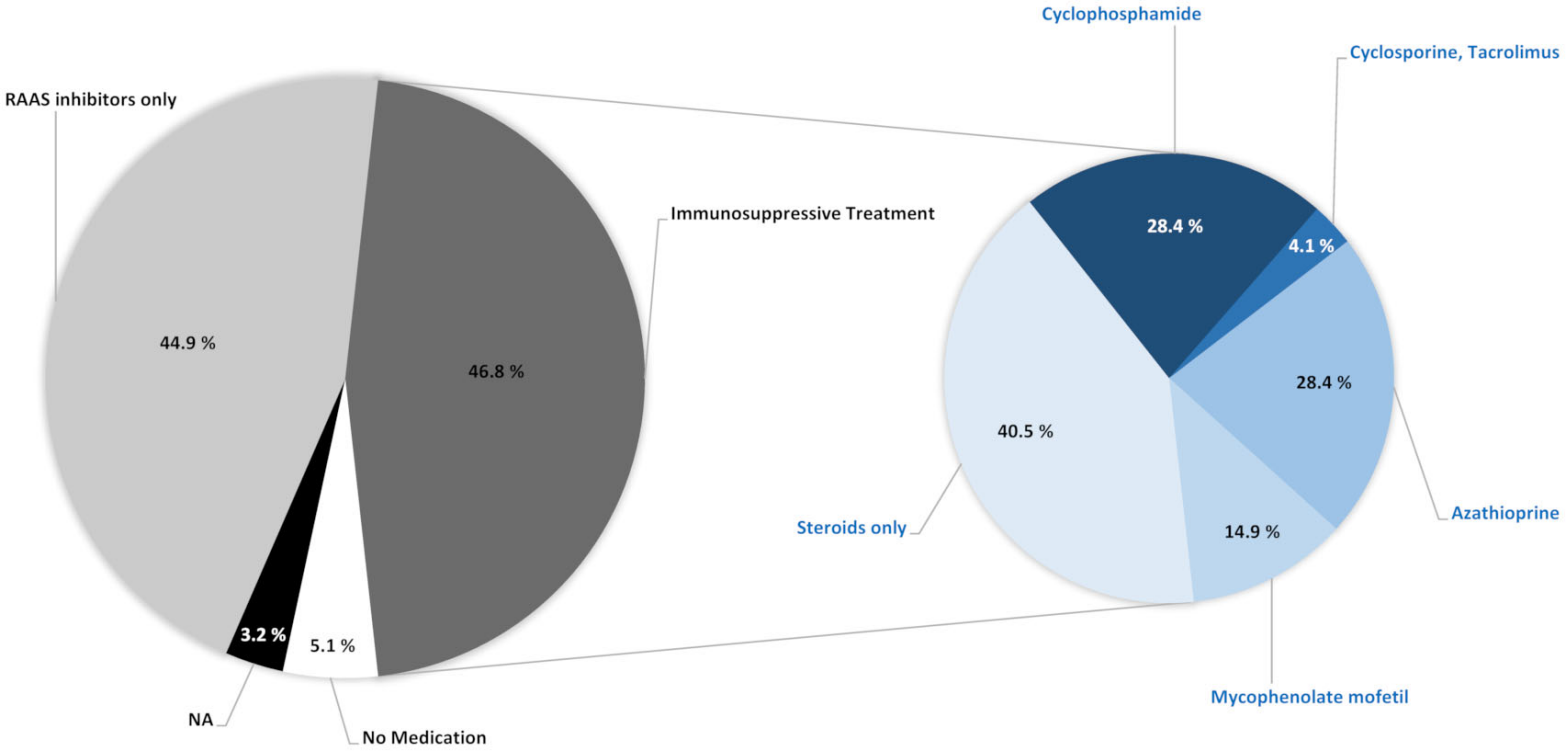
Overall therapy since diagnosis. Values are expressed as percentage of patients receiving specific therapies. RAAS, Renin-Angiotensin-Aldosterone System; NA, data not available.

Administered therapies are displayed in **Figure 4**. Overall, the treatment regimen of 86.7% of the patients included inhibitors of the RAAS; half of the patients without RAAS inhibitor therapy had received a diagnosis of IgA nephropathy before 1990 (market introduction of RAAS inhibitors). In 44.9% of patients, therapy was based exclusively on RAAS inhibitors whereas 46.8% of patients received immunosuppressive therapy in addition to RAAS inhibitor treatment. In nearly all (95.7%) patients, the immunosuppressive regimen included oral and/or intravenous glucocorticoids with 40.5% of these patients receiving glucocorticoid monotherapy. The most frequently used non-steroid therapies were cyclophosphamide and azathioprine, followed by mycophenolate mofetil and finally by calcineurin inhibitors.

Overall, remission rate was 56.3% with 34.1% of all patients fulfilling criteria for complete remission and 22.2% reaching partial remission respectively (**Table 2**). At the last follow-up, mean serum creatinine was 167.4 µmol/l with a mean eGFR according to CKD-EPI of 55.2 ml/min/1.73 m^2^ and mean proteinuria of 0.8 g/d. Mean systolic and diastolic blood pressure values were 129.5 mmHg and 79.0 mmHg respectively. A total of 43.0% of patients progressed to ESKD during follow-up, whereas 55.6% of patients developed ESKD within 7 years of follow-up. Among patients not reaching ESKD, 7.9% had a doubling of serum creatinine value at some point during follow-up. Nearly a third of the patients (31.6%) received a kidney transplant. One-fifth (18.8%) of the transplanted patients developed recurrence of IgA nephropathy within a median follow-up time of 79.7 (10.8 – 457.1) months. Graft rejections occurred in 11.1% of transplanted patients. The mortality rate in the total cohort was 8.9% within a median follow-up time of 100.0 (0.2-457.1) months.

**Table 2:** Patient outcome.

Blood pressure (mmHg) at last follow-up (n=138)	
- systolic	129.5 ± 14.9
- diastolic	79.0 ± 11.5
Serum creatinine (µmol/l) at last follow-up (n=142)	167.4 ± 144.0
Estimated glomerular filtration rate* (ml/min/1.73 m^2^) at last follow-up	55.2 ± 28.0
Proteinuria^+^ (g/d) at last follow-up (n=144)	0.8 ± 1.7
Remission after treatment^#^ (%)	
- partial remission	22.2
- complete remission	34.1
- no response	43.7
Acute kidney injury at any time-point after diagnosis	12.0
Doubling of serum creatinine (without ESKD) (%)	7.9
End-stage kidney disease (%)	43.0
End-stage kidney disease within 7 years (%)	55.6
Kidney transplantation (%)	31.6
- graft rejection rate (%)	11.1
- IgAN recurrence after transplantation (%)	18.8
Death (%)	8.9
Median follow-up time (months)	100.0 (0.2-457.1)

Values are expressed as means with standard deviation, medians with ranges as indicated or proportions of patients. ESKD: end-stage kidney disease. *according to Chronic Kidney Disease Epidemiology Collaboration 2009 formula. ^+^measured from adequately collected 24h-urine collection if available or estimated from urinary protein-creatinine ratio. Acute kidney injury as defined by Kidney Disease Improving Global Outcomes Guidelines 2012. ^#^ defined as best therapy response occurring within one year after first treatment line.

### 3.4 Predictors of immunosuppressive therapy use

The comparison between patients treated with and without immunosuppressive therapy showed several differences (**Table 3**). Immunosuppressive therapy was more frequently prescribed to patients with nephrotic syndrome and higher proteinuria level, higher hematuria intensity, lower eGFR and in the presence of extrarenal symptoms. In addition, patients with histological findings including presence of crescents and higher immunoglobulin A and C3 staining intensity on kidney biopsy were more likely to receive immunosuppressive treatment. In contrast, age did not significantly affect the decision to apply immunosuppressive therapy. Interestingly, degrees of IFTA were higher at baseline in patients finally treated with immunosuppression.

**Table 3:** Group comparison according to use of immunosuppressive therapy.

Patient characteristics at baseline	Immunosuppressive therapy	No immuno-suppressive therapy	T (df)	χ2 (df)	P value
	(N = 74)	(N = 76)			
Age (years)	52.9 ± 14.6	53.3 ± 15.0	0.181 (148)		0.857
Female (%)	29.7	25.0		0.422 (1)	0.584
Systolic BP (mmHg)	138.9 ± 23.8	138.3 ± 20.5	-0.155 (123)		0.877
Diastolic BP (mmHg)	85.6 ± 13.8	82.9 ± 14.8	-1.058 (123)		0.292
eGFR^*^ (ml/min/1.73 m^2^)	49.9 ± 31.0	60.9 ± 29.7	2.008 (132)		**0.039**
Proteinuria 24h^+^ (g/d)	3.2 ± 2.3	1.6 ± 1.6	-4.599 (109.852)		**<0.001**
Nephrotic Syndrome (%)	25.0	1.5		15.248 (1)	**<0.001**
Hematuria (0-4)	3.26 ± 1.26	2.68 ± 1.62	-2.237 (115.123)		**0.027**
Extrarenal symptoms^#^ (%)	0.0	9.9		–	
Crescents (% glomeruli)	9.4 ± 15.0	2.3 ± 8.5	**-**3.216 (90.578)		**0.002**
Presence of crescents (%)	54.9	21.9		16.616 (1)	**<0.001**
Tubular atrophy/Interstitial Fibrosis (1-3)	1.74 ± 0.89	1.32 ± 0.63	-3.116 (114.013)		**0.002**
IgA staining intensity (0-4)	2.67 ± 0.72	2.31 ± 0.89	-2.642 (134)		**0.009**
IgG staining intensity (0-4)	0.75 ± 0.72	0.51 ± 0.68	-1.980 (132)		**0.050**
C3 staining intensity (0-4)	2.04 ± 0.92	1.59 ± 0.93	-2.327 (91)		**0.022**

Values are expressed as means with standard deviation or as proportions of patients. P-values according to student’s t-test or Chi square-test as appropriate. T(df): T statistic (degree of freedom). χ2 (df): Pearson Chi-Square statistic (degree of freedom). ^*^eGFR: estimated glomerular filtration rate according to Chronic Kidney Disease Epidemiology Collaboration 2009 formula. ^+^measured from adequately collected 24h-urine collection if available or estimated from urinary protein-creatinine ratio. ^#^ not formally tested due to distribution and low number of events. P-values ≤ 0.05 are marked in bold.

### 3.5 Predictors of remission status

The comparison of baseline parameters between the patient subgroups according to remission status (within one year after first treatment regimen) is shown in **Table 4**. At the time of first diagnosis, as expected, macrohematuria, lower systolic blood pressure, higher baseline eGFR, lower proteinuria level and less IFTA were predictors of remission. Nephrotic syndrome did not significantly affect the remission outcome. When separated according to decades, lack of therapy response occurred in a high proportion of patients before 1990, whereas this rate remained stable at roughly 30% thereafter; similarly, overall remission rates increased after 2000. However, the number of patients diagnosed before 2000 was low (**Supplementary Table 1** and **Supplementary Figure 1**).

**Table 4:** Group comparison according to remission status.

Patient characteristics at baseline	Remission^#^	No remission	T (df)	χ2 (df)	P value
	(n = 71)	(n = 55)			
Age (years)	53.4 ± 14.0	55.0 ± 15.2	0.590 (124)		0.556
Female (%)	29.6	20.0		1.500 (1)	0.302
Systolic BP (mmHg)	135.3 ± 20.5	145.5 ± 23.4	2.387 (105)		**0.019**
Diastolic BP (mmHg)	82.3 ± 14.9	87.5 ± 13.2	1.860 (105)		0.066
eGFR* (ml/min/1.73 m^2^)	60.6 ± 29.3	40.3 ± 27.2	-3.771 (113)		**<0.001**
Proteinuria^+^ (g/d)	2.2 ± 1.9	3.2 ± 2.3	2.335 (108)		**0.021**
Nephrotic Syndrome (%)	11.1	19.5		1.420 (1)	0.263
Macrohematuria (%)	39.1	18.5		6.119 (1)	**0.017**
Crescents (% glomeruli)	5.9 ± 10.9	6.7 ± 16.5	0.304 (108)		0.762
Presence of crescents (%)	42.3	35.3		0.602 (1)	0.459
Tubular atrophy/Interstitial Fibrosis (1-3)	1.39 ± 0.65	1.83 ± 0.94	2.788 (76.099)		**0.007**
IgA staining intensity (0-4)	2.53 ± 0.84	2.51 ± 0.74	- 0.114 (113)		0.910
IgG staining intensity (0-4)	0.76 ± 0.71	0.54 ± 0.72	- 1.572 (111)		0.119
C3 staining intensity (0-4)	1.78 ± 0.85	2.10 ± 0.94	1.511 (74)		0.135

Values are expressed as means with standard deviation or as proportions of patients. P-values according to student’s t-test or Chi square test as appropriate. T(df): T statistic (degree of freedom). χ2 (df): Pearson Chi-Square statistic (degree of  freedom). *eGFR: estimated glomerular filtration rate according to Chronic Kidney Disease Epidemiology Collaboration 2009 formula. ^+^ measured from adequately collected 24h-urine collection if available or estimated from urinary protein-creatinine ratio. ^#^defined as any remission occurring within one year after first treatment line. P-values ≤ 0.05 are marked in bold.

### 3.6 Predictors of progression to end-stage kidney disease

Patients who progressed to ESKD were on average older and had higher blood pressure, lower eGFR and higher proteinuria level at baseline as compared to non-progressors (**Table 5**). In addition, patients reaching ESKD more frequently exhibited nephrotic syndrome at the time of diagnosis, while clinical remission had occurred less frequently. Overall, 13.3% of patients with proteinuria <1g/d at baseline developed ESKD in this cohort. Histologically, more extensive IFTA and crescents were present at baseline in progressors.

**Table 5:** Group comparison according to progression status.

Patient characteristics at baseline and remission status	ESKD	No ESKD	t (df)	χ^2^ (df)	P value
	(N = 68)	(N = 90)			
Age (years)	57.2 ± 13.5	50.7 ± 15.2	- 2.790 (156)		**0.006**
Female (%)	22.1	30.0		1.252 (1)	0.281
Systolic BP (mmHg)	146.0 ± 26.4	134.5 ± 18.4	- 2.519 (61.260)		**0.014**
Diastolic BP (mmHg)	89.0 ± 14.3	81.6 ± 13.6	- 2.824 (125)		**0.006**
eGFR* (ml/min/1.73 m^2^)	41.1 ± 28.8	64.2 ± 28.5	4.535 (134)		**<0.001**
Proteinuria^+^ (g/d)	3.4 ± 2.0	1.8 ± 1.9	- 4.553 (127)		**<0.001**
Nephrotic syndrome (%)	20.9	7.5		4.711 (1)	**0.042**
Crescents (% glomeruli)	8.8 ± 16.9	3.7 ± 8.8	- 1.874 (56.254)		0.066
Presence of crescents (% patients)	52.5	28.9		8.538 (1)	**0.004**
Tubular atrophy/Interstitial Fibrosis (1 – 3)	1.88 ± 0.916	1.26 ± 0.560	-4.511 (82.136)		**<0.001**
IgA staining intensity (0-4)	2.55 ± 0.81	2.43 ± 0.84	- 0.838 (138)		0.403
IgG staining intensity (0-4)	0.64 ± 0.68	0.59 ± 0.72	- 0.440 (137)		0.660
C3 staining intensity (0-4)	1.95 ± 1.12	1.72 ± 0.83	- 1.061 (48.342)		0.294
Overall remission^#^ (%)	34.0	78.1		24.809 (1)	**<0.001**
Partial remission^++^ (%)	23.9	38.5		1.702 (1)	0.280

Values are expressed as means with standard deviation or as proportions of patients. P-values according to student’s t-test or chi square test as appropriate. T(df): T statistic (degree of freedom). χ^2^ (df): Pearson Chi-Square statistic (degree of freedom). ESKD: End stage kidney disease. *eGFR: estimated glomerular filtration rate according to Chronic Kidney Disease Epidemiology Collaboration 2009 formula. ^+^measured from adequately collected 24h-urine collection if available or estimated from urinary protein-creatinine ratio. ^#^defined as best therapeutic response at any time during follow-up including partial and complete remission. ^++^defined as best therapeutic response occurring at any time during follow-up. P-values ≤ 0.05 are marked in bold.

## 4 Discussion

The goal of this study was to perform a detailed review of all patients with IgA nephropathy treated at our Swiss tertiary center focusing on disease characteristics, treatment practices and outcome as well as to evaluate potential predictors of therapeutic choices and patient outcome. The main findings were the following: i) a large spectrum of age-dependent clinical manifestations, ii) use of immunosuppressive therapy, particularly non-steroid immunosuppressive therapy, in an important part of patients, and iii) progression to ESKD in a comparatively high proportion of patients.

First, in this patient cohort of mainly European ancestry exhibiting an expected sex distribution, a wide range of clinical presentations was noted including isolated urine abnormalities, nephrotic syndrome, chronic kidney function impairment and AKI as previously reported (5). However, chronic kidney function impairment was by far the most common clinical presentation. This may be due to the older patient age and the advanced stage of disease at diagnosis (i.e. higher proportion of patients with moderate to severe IFTA) as compared to other cohorts (23). Indeed, in this cohort, disease presentation varied considerably according to age. As expected, presentation with macrohematuria as sole disease manifestation was more frequent in younger adults decreasing in frequency with age. In addition, in this cohort, younger adults more often presented with asymptomatic urine abnormalities. Isolated microhematuria was not noted since it usually does not represent an indication for kidney biopsy in Switzerland. Interestingly, in this cohort, the frequency of extrarenal symptoms pointing to a diagnosis of Henoch-Schönlein purpura was not significantly different among different age groups whereas a usually higher frequency is described in younger patients (25). However, this might be explained by the exclusion of children from this cohort and more frequent secondary etiologies in adults including drugs, infection and cancer becoming more prevalent with age. Lastly, in this cohort, nephrotic syndrome classically described as an uncommon clinical presentation in IgA nephropathy, was noted at baseline in roughly 10% of patients and more frequently so in women. This is in contrast to data from Chinese patients with IgA nephropathy indicating more severe clinical presentation including proteinuria level in male patients (26). The reasons for this discrepancy are not clear. Although spontaneous remissions have been described in the setting of nephrotic syndrome in IgA nephropathy and to be more frequent in female patients according to a Korean study, in our cohort, the presence of nephrotic syndrome was more often observed among patients subsequently treated with immunosuppressive therapy (27).

Second, almost half of the included patients had received immunosuppressive treatment for IgA nephropathy. In comparison, 19-25% of patients had received immunosuppressive treatment in the German CKD cohort and a Chinese glomerulonephritis registry (28, 29). Although this proportion might therefore appear high, a comparable proportion of patients had received immunosuppressive therapy in the VALIGA cohort. This might be explained by the major contribution of Italian centers to the last-mentioned cohort with traditional comparatively higher use of immunosuppressive therapy in the treatment of IgA nephropathy (18, 19, 23). Furthermore, however, more than half of the patients in our cohort had received a non-steroid-based immunosuppressive regimen, which corresponds to a clearly higher proportion of patients than in the VALIGA cohort (23). Among the employed therapies, cyclophosphamide and azathioprine were the most common. As expected, higher proteinuria represented a major predictive factor for the decision to use immunosuppressive therapy in these patients. Additionally however, 28.9% of the patients with proteinuria <1g/d at presentation had received immunosuppression for the treatment of IgA nephropathy. It can be speculated that the more severe presentation at baseline in our cohort including higher proteinuria levels might have favored the use of immunosuppressive therapies although there were no prevailing internal pre-specified treatment criteria. In addition, as referral center, therapy-refractory disease or patients pre-treated with non-immunosuppressive and steroid-based regimens may have been disproportionally represented. The frequent use of azathioprine might also be explained by the participation in an international randomized-controlled trial investigating the effect of azathioprine in the treatment of IgA nephropathy (30). Lastly, the large majority of patients in this cohort (85.4%) had been diagnosed before publication of the Stop-IgAN trial results (16). The coverage by RAAS inhibitor treatment in our cohort was large and comparable to other cohorts taking into account the small proportion of patients treated before the advent of this drug class.

Third, a high rate of progression was noted in our cohort, with over half of the patients reaching ESKD within 7 years of follow-up, 43% of the patients during a mean follow-up time of 8.3 years. In contrast, 18.8% of patients in the European VALIGA cohort progressed to ESKD during a median follow-up time of 7 years, 12.6% of patients in the German CKD cohort over 6.5 years, while 34% in a Norwegian cohort reached a combined end-point of ESKD or death within 8 years (28, 31, 32). Based on the French REIN registry, IgA nephropathy represented roughly 7% of patients among newly diagnosed ESKD in recent years although analysis of progression rates were not aim of the study (33). Similarly, lower rates of progression are reported among patient cohorts of non-European ancestry (29). This finding might be explained by the rather advanced presentation at baseline in our cohort including lower eGFR, higher proteinuria, higher blood pressure and greater extent of chronic interstitial lesions on kidney biopsy (23, 28, 29, 32). Indeed, in our cohort, lower eGFR and higher proteinuria at presentation were confirmed as predictors of progression to ESKD. In line with this hypothesis, a similarly poor outcome has been reported recently in a UK cohort of patients with comparably severe clinical presentation (34). In addition, the comparatively older age of patients in our cohort might have contributed to worse outcomes (23, 29, 32). Finally, the inclusion of patients treated at a tertiary clinical center might have led to selection of more severe cases. Indeed, the clinical presentation with macroscopic hematuria in our cohort was much less frequent than described previously (5). It is further important to note that 13.3% of patients presenting with proteinuria <1g/d in this cohort progressed to ESKD during follow-up extending recent findings from the UK RaDaR cohort correlating time-averaged proteinuria to development of ESKD (34).

Interestingly, the degree of chronic changes on the initial kidney biopsy (i.e. IFTA) did not predict utilization of immunosuppressive therapy in this cohort. In contrast, an opposite trend was found with patients receiving an immunosuppressive treatment having higher degrees of IFTA at baseline. The reasons for this finding are unclear. The degree of IFTA did not represent an exclusion criterion in the randomized-controlled trial evaluating azathioprine mentioned above (30). However, no difference was observed regarding IFTA severity in patients treated with azathioprine and the rest of the cohort. Other factors identified as predictors of the use of immunosuppression in patients with IgA nephropathy in our study such as proteinuria level, nephrotic syndrome and the presence of crescents mirror the findings of a recent survey (21).

Limitations of this analysis have to be considered. Firstly, the retrospective design precludes reconstruction of therapy choices although significant differences among treatment groups in this cohort point to factors influencing therapeutic decisions. Secondly, analysis from a single center cohort in a tertiary center may have led to selection bias and reduced generalizability. Thirdly and similarly, in this patient cohort of mainly European ancestry, results have to be interpreted within this context. Fourthly, since the historical eponym Berger’s disease has not been included in the search strategy, very few patients with IgA nephropathy might have been missed in the analysis. However, the exclusive use of this eponym after 2008 without mention of IgA deposits in the histological description seems unlikely. Fifthly, in this analysis, univariate analysis of predictors has been performed, while data of all performed comparisons are shown. Sixthly, our patient cohort is of limited sample size. However, despite this, significant predictors of therapy and outcome could be identified. Lastly, due to the majority of patients having been diagnosed before the introduction of the Oxford classification, MEST scores were available only for a minority of patients.

In conclusion, at the dawn of a new therapeutic era for patients with IgA nephropathy (35), this retrospective cohort analysis from 1980–2016 gives detailed insight into clinical and histological characteristics and outcome of patients with IgA nephropathy from a Swiss tertiary center as well as treatment practices and potential predictors of outcome and therapy.

## Data Availability

The original contributions presented in the study are included in the article/**Supplementary Material**. Further inquiries can be directed to the corresponding author.
