# Supplementary material for: Treatment practices, characteristics and outcome of immunoglobulin A nephropathy – a Swiss single center experience
Source: Front Nephrol. 2026 Feb 27;6:1648950. doi: 10.3389/fneph.2026.1648950 (PMC12982110; doi:10.3389/fneph.2026.1648950)
Supplement: Supplementary file 1 [file DataSheet1.pdf]

Supplemental Table 1. Remission status according to decade.

**Decade**	**1980 – 1989**	**1990 – 1999**	**2000 – 2009**	**From 2010**
	**(N = 10)**	**(N = 26)**	**(N = 58)**	**(N = 63)**
No response	80.0 %	30.8 %	27.6 %	30.2 %
Partial remission	0.0 %	7.7 %	17.2 %	14.3 %
Complete remission	0.0 %	38.5 %	39.7 %	33.3 %
