# Supplementary figures and images for: Treatment practices, characteristics and outcome of immunoglobulin A nephropathy – a Swiss single center experience
Source: Front Nephrol. 2026 Feb 27;6:1648950. doi: 10.3389/fneph.2026.1648950 (PMC12982110; doi:10.3389/fneph.2026.1648950)

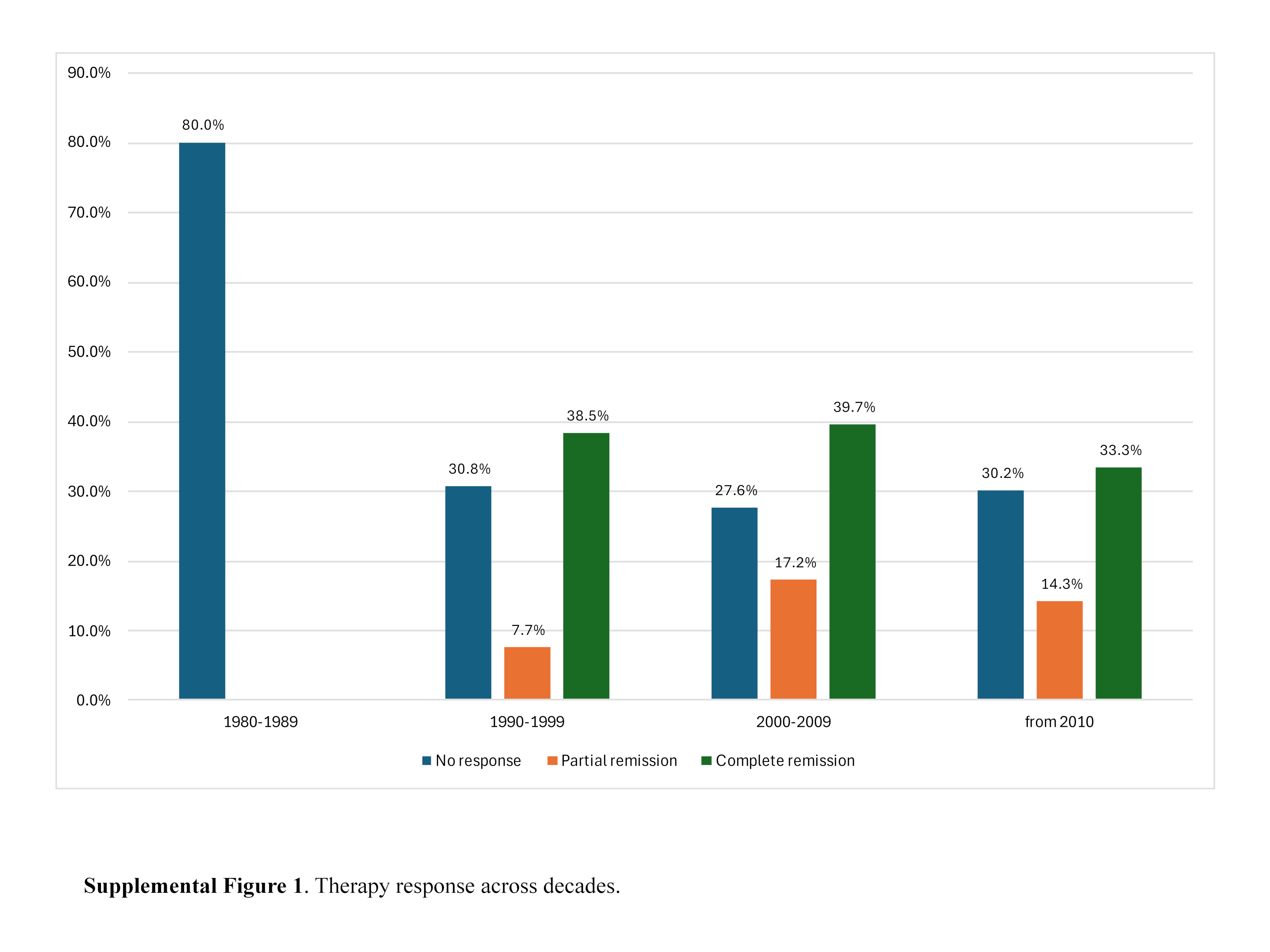

Supplement: Supplementary file 2 [file Image1.tif]
